# Unraveling the Effects of Co-Crystallization on the UV/Vis Absorption Spectra of an *N*-Salicylideneaniline Derivative. A Computational RI-CC2 Investigation

**DOI:** 10.3390/molecules25194512

**Published:** 2020-10-01

**Authors:** Jean Quertinmont, Tom Leyssens, Johan Wouters, Benoît Champagne

**Affiliations:** 1 Unité de Chimie Physique Théorique et Structurale, Namur Institute of Structured Matter, University of Namur, 61 rue de Bruxelles, B-5000 Namur, Belgium; jean.quertinmont@unamur.be (J.Q.); johan.wouters@unamur.be (J.W.); 2 Institute of Condensed Matter and Nanosciences, Université Catholique de Louvain, 1 Place Louis Pasteur, B-1348 Louvain-La-Neuve, Belgium; tom.leyssens@uclouvain.be

**Keywords:** co-crystal, anil molecular switch, UV-vis absorption, coupled cluster, embedding schemes

## Abstract

This work aims at unraveling the effects of co-crystallization on the optical properties of an *N*-salicylideneaniline-derived molecular switch transforming between an enol and a keto form. This is achieved by way of a two-step multi-scale method where (i) the molecular geometry and unit cell parameters are optimized using a periodic boundary conditions density functional theory method and (ii) the optical properties are computed for a selection of clusters embedded in an array of point-charges that reproduce the crystal field electronic potential. The optical properties (vertical excitation energies and oscillator strengths) are obtained at the RI-CC2/def2-TZVPD level of approximation. This method allows us to decompose the effects of co-crystallization into (i) indirect effects, the geometry changes of the chromophore due to crystal packing with the coformer, and (ii) direct ones, the polarization due to the interacting coformer and to the crystal field. For the former effects, variations of a crucial torsion angle lead to modification of the π-conjugation and therefore to the decrease or increase of the excitation energies. About the latter, they are antagonistic: (i) the coformer is not directly involved in the excitations but its polarization decreases the excitation energies while (ii) the crystal field has the opposite effect. For the co-crystals with succinic and fumaric acids, combining these direct and indirect effects leads to a hypsochromic shift of the first absorption band with respect to the reference crystal, in agreement with experimental data.

## 1. Introduction

**Scheme 1:**
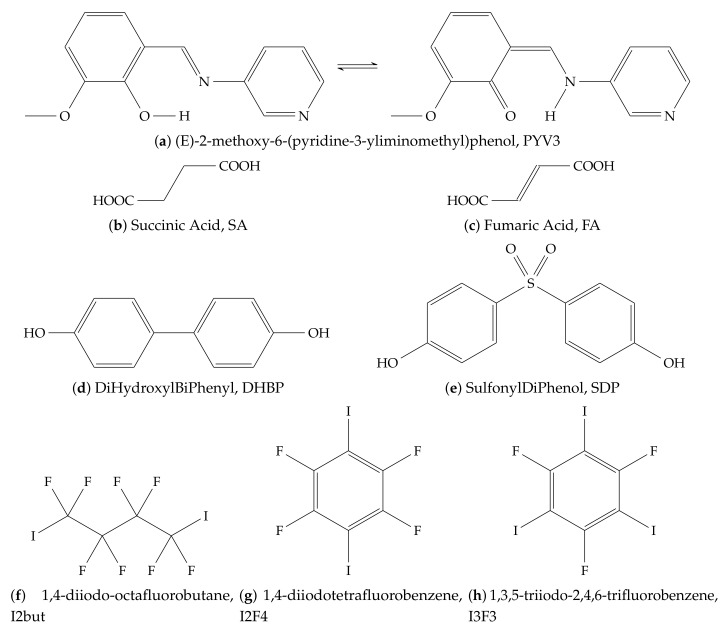
Structures of PYV3 and its coformers, (**a**) anil and its enol–keto equilibrium; coformers interacting by H-bond (**b**–**e**) or X-bond (**f**–**h**).

**Figure 1:**
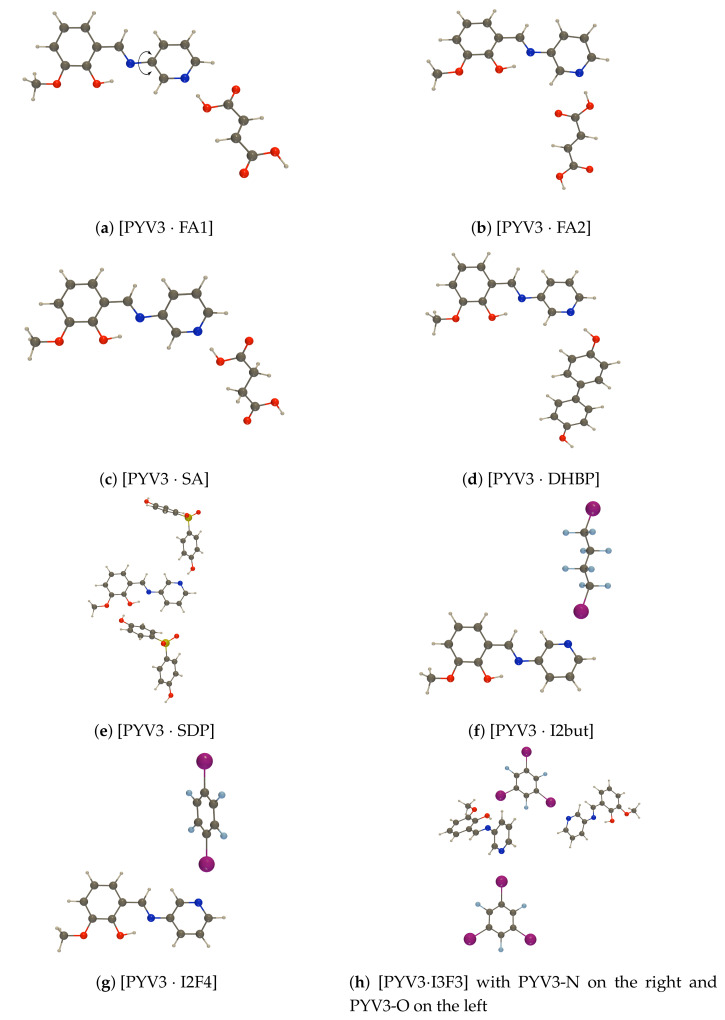
Representation of the PYV3-coformer synthons as extracted from the co-crystal structure optimized at the PBC/PBEsol0/6-31G(d,p)/I(LANL2DZ) level of approximation. Here, the enol form was considered for all co-crystals. The corresponding references to the X-ray diffraction structures are also provided. In the case of (**a**), an arrow has been added to define the pyridine torsion angle.

Co-crystallization can be used to tune the properties of molecular switches: for example, a *N*-salicylideneaniline (anil), which equilibrates between an enol (E) and a keto (K) form becomes photochromic upon co-crystallization with two different coformers: succinic acid (SA) or fumaric acid (FA) [1]. The coformer can have effects at multiple levels: changing the thermodynamics and/or the kinetics of the tautomeric equilibrium and modifying its electronic properties such as the UV/Vis absorption or the excited state dynamics. These are generally related to a change of the geometry as well as to steric hindrance and confinement effects. The effects on the relative energy on the geometry have already been addressed in Ref. [2]. Focusing now on the effects on the UV/Vis absorption spectra, a series of hydrogen bond and halogen bond co-crystals of (E)-2-methoxy-6-(pyridine-3-yliminomethyl)phenol, PYV3, are studied (Scheme 1 for its structure, the enol/keto equilibrium, and the coformers). The synthons of the enol forms of the co-crystals are given in Figure 1 ([PYV3 · FA1] [1], [PYV3 · FA2] [1], [PYV3 · SA] [1], [PYV3 · DHBP] [3,4], [PYV3 · SDP] [3,4], [PYV3 · I2but] [5], [PYV3 · I2F4] [5,6], and [PYV3·I3F3] [5]). Note that in the case of the fumaric acid (FA) coformer, there are two polymorphic co-crystals named PYV3 · FA1 and PYV3 · FA2, where the former is isostructural to PYV3 · SA. Based on those, we define the “monomer” of the co-crystal as a single anil molecule (either in its enol or keto form) and the “heteromers” as the anil and its directly interacting coformer(s). In our nomenclature, PYV3 [PYV3 · SA] stands for the monomer of the co-crystal of PYV3 with succinic acid, i.e., the PYV3 molecule in the geometry of its co-crystal with SA. Similarly, PYV3 + SA [PYV3 · SA] is the heteromer of the same co-crystal, i.e., one PYV3 and one SA molecule as in the PYV3 · SA co-crystal (synthon c of Figure 1). The objective of this work is to study the effects of co-crystallization on the optical properties: the excitation energies, the oscillator strengths, the electronic transitions, and the UV/Vis absorption spectra.

The proposed methodology consists of combining firstly periodic boundary conditions (PBC) density functional theory (DFT) method to optimize both the molecular geometry and unit cell parameters of the crystal and secondly an embedding method to compute the optical properties. This methodology allows us to decompose the effects of the (co-)crystal surrounding into direct and indirect effects. The latter originates from the fact that upon (co-)crystallization, the geometry of the chromophore is modified, which leads to variations of the optical properties. On the other hand, the former originates from the effect of the surrounding polarization on the ground and excited-state wavefunctions, and therefore influences the absorption spectra. The calculation of the optical properties is achieved by extracting a monomer or heteromer from the crystal structure and then embedding it in an array of point-charges fitted to reproduce the full Coulombic potential of the crystal.

The array is computed by the Ewald program [7,8]. It uses three zones: the first one contains the molecule(s) on which a quantum treatment is performed on, zone 2 is made of unmodified point-charges, while the point-charges of zone 3 are fitted to reproduce the Ewald potential. The three zones are user-defined. The calculations of the optical properties are done at the coupled-cluster approximate doubles (CC2) level of approximation [9] in combination with the resolution of the identity (RI) approximation [10,11]: RI-CC2. This method presents the advantage of being less computationally demanding than the more accurate coupled cluster singles and doubles (CCSD) model. While time-dependent DFT (TD-DFT) could have been used, it poses significant additional questions: which exchange correlation functional (XCF) to use? Can a single functional be accurate for both excitation energies and oscillator strengths? Or for both the anil and its coformers? Thus, to get away from those potential issues, RI-CC2 was selected. Yet, this is not a perfect solution, as it comes with its own issues, namely how approximate are the doubles compared to CCSD? This particular topic, as well as the choice of the basis set are therefore tackled in the next section. Despite that, it was already shown that CC2 performs well for both the excitation energies and the oscillator strengths [9,12,13,14,15,16]. Some of the tools used to analyze the excited states are the NTOs, which describe the electronic transitions in a more “natural” fashion than with MOs [17,18]. Their properties and methods of calculations are described in the following section. This three-step scheme has already been successfully applied to the evaluation of different types of properties in the solid state [19,20,21,22], including in the case of anil derivatives [20,21].

## 2. Results and Discussion

### 2.1. Effects of the Geometry

**Figure 2:**
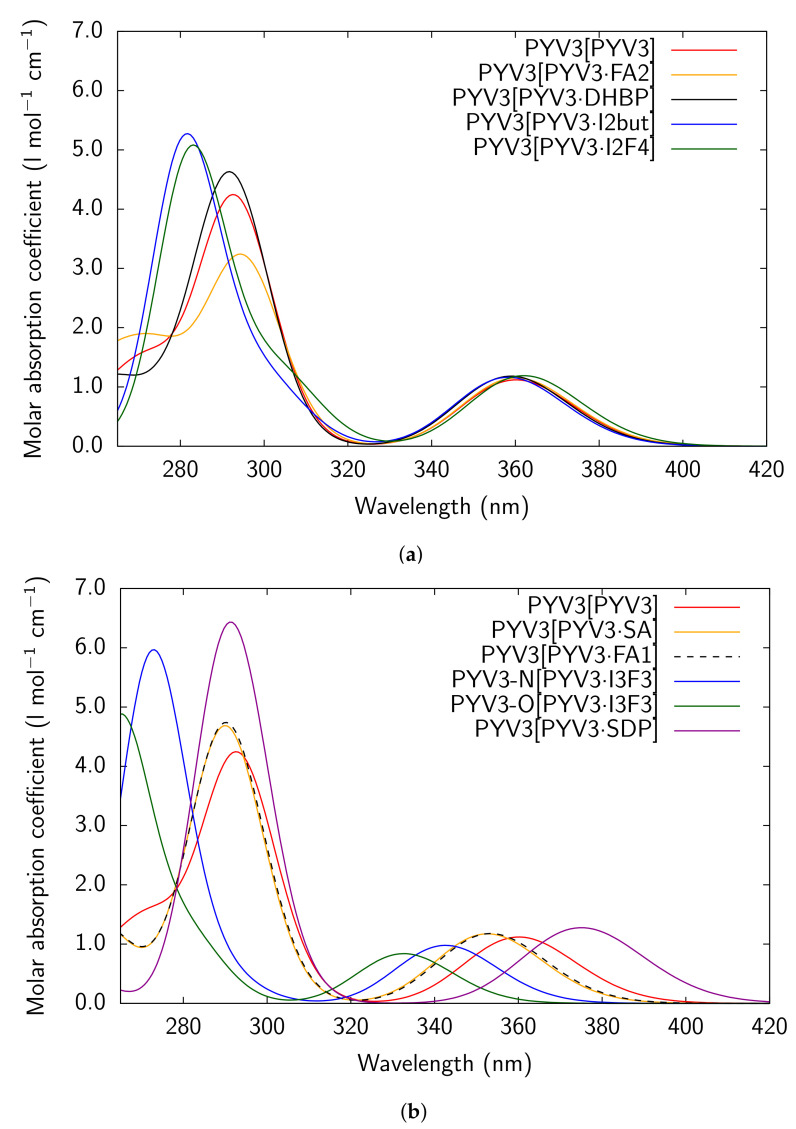
RI-CC2/def2-TZVPD UV/Vis absorption spectra of isolated monomers of all-enol crystals (PYV3 [PYV3 · XXX]). For clarity, the spectra have been splitted in two parts, see (**a**) and (**b**).

The effects of co-crystallization are multifold. Ref. [2] analyses some of those: the changes in the relative energy of the tautomers, in the geometries, and in the atomic charges distributions. This work used the crystal geometries that have been optimized in Ref. [2]. The root mean square deviation for the atomic positions of the PYV3 co-crystals compared to PYV3 range from 0.131*Å*–1.637*Å* for the enol forms and from 0.037*Å*–1.630*Å* for the keto ones. These large values are associated with the significant variations of the torsion angle of the pyridine. More details can be found in Ref. [2]. The characteristics of the first excitation of the isolated monomers of PYV3 (PYV3 [PYV3]) and of its co-crystals (PYV3 [PYV3 · XXX]) are given Table 1. For the enol forms, the PYV3 · FA2, PYV3 · DHBP, and PYV3 · I2F4 co-crystals are barely affected, with variations of up to 2 nm for the first excitation wavelengths. The corresponding oscillator strength variations do not exceed 6. Figure 2a shows those simulated spectra, highlighting the overlap of the first bands (fully characterized by the first excitation). The second bands feature two excitations. They are more affected by the co-crystallization than the first ones. Indeed, changes in both excitation energies and oscillator strengths result in (i) similarly positioned but less (PYV3 · FA2) or more (PYV3 · DHBP) intense bands or (ii) blue-shifted bands (PYV3 · I2but and PYV3 · I2F4), see also Table A4. Compared to PYV3, both are red-shifted by ∼8 nm but because the 2nd excitation is much less intense in favor of the 3rd one, the actual band maximum is blue-shifted by about 10 nm. The second set of co-crystals (Figure 2b), co-crystals PYV3 · SA, ·FA1, and ·I3F3, display larger first excitation energies with blue shifts ranging from 7nm–27nm. As expected from Ref. [2] that highlights the isostructurality of the PYV3 · SA and PYV3 · FA1 co-crystals, their spectra almost perfectly overlap. Both of their bands are slightly blue-shifted ( 2 nm) with respect to PYV3 and thanks to a larger oscillator of the 2nd excitation, the second band is more intense. The largest variations are obtained for the PYV3·3F3 monomers: the first excitation of PYV3-N [PYV3 · I3F3] is shifted by −18 nm and is less intense, by 13, while that of PYV3-O [PYV3 · I3F3] by −27 nm and 25. Similarly to PYV3 · I2but and PYV3 · I2F4, the 2nd bands of PYV3-N [PYV3 · I3F3] and PYV3-O [PYV3 · I3F3] are strongly blue-shifted and more intense than PYV3. In these cases, the 2nd and 3rd excitations are both blue-shifted in addition to a strong decrease of oscillator strength of the 2nd excitation and an even stronger increase of the 3rd one (Table A4). Lastly, SDP is the only example of a strong decrease of the excitation energy, i.e., a red-shift of the first excitation wavelength by 15 nm. Like for its second band, it is uniquely defined by the second excitation of similar energy, but stronger oscillator strength, than PYV3.

**Figure 3:**
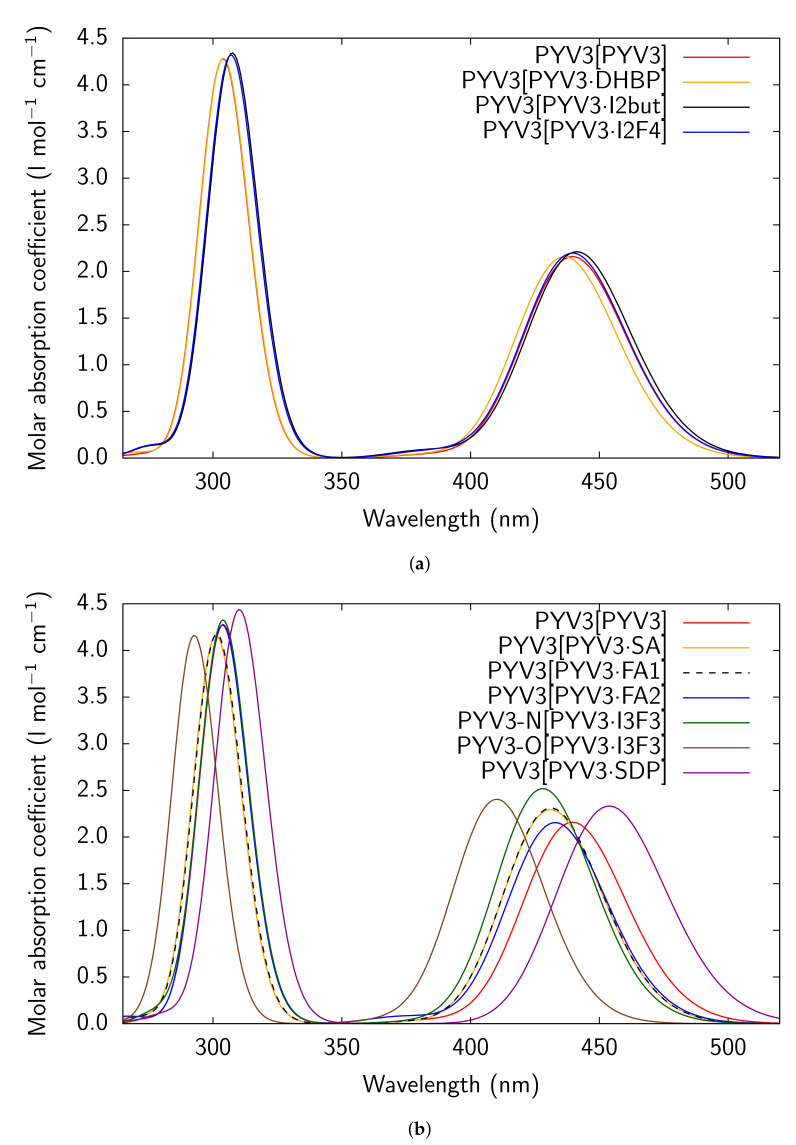
RI-CC2/def2-TZVPD UV/Vis absorption spectra of isolated monomers of all-keto crystals (PYV3 [PYV3 · XXX]). For clarity, the spectra have been splitted in two parts, see (**a**) and (**b**).

For the keto forms, the first excitations of the PYV3 molecule are barely affected by co-crystallization in the case of the PYV3 · DHBP, PYV3 · I2but, and PYV3 · I2F4 co-crystals (Figure 3a, with shifts ranging from -3nm–2nm and oscillator strength variations of 0–2). Similarly, the third excitations, i.e., the second bands, are barely affected: shifts ranging from 0nm–4nm and oscillator strength variations of 0–1 (Table A5). These UV/Vis absorption spectra are given in Figure 3a. The remaining co-crystals, PYV3 · SA, ·FA1, ·FA2, ·I3F3, and SDP, are shown in Figure 3b. Again, PYV3 · SA and PYV3 · FA1 have identical spectra where the first band is blue-shifted by 8 nm but the second one is barely changed ( ∼2 nm shift and ∼2 less intense). PYV3 · FA2 differs from PYV3 only by the position of the first band, shifted by −7 nm. The PYV3 chromophore sees the largest increase of the first excitation energy corresponding to Δλ of 12 nm for PYV3-N [PYV3 · I3F3] and 29 nm for PYV3-O [PYV3 · I3F3]. They also display stronger absorptions ( 17 for PYV3-N and 11 for PYV3-O). Their second bands are less affected. Indeed, PYV3-N [PYV3 · I3F3] is not shifted while IPYV3-O [PYV3 · I3F3] is shifted by −11 nm (less than half of the 1st band). Their intensities are on par with that of PYV3 (-3–1). Like for the enols, PYV3 · SDP is the only case with a significant decrease of the 1st excitation energy, or Δλ=14nm, also accompanied by a stronger absorption with respect to PYV3 (by 8 ). The 2nd band (3rd excitation) is also red-shifted, but by a smaller amount,  6 nm.

The first excitation shifts of PYV3-O [PYV3 · I3F3] ( −28 nm) and PYV3 [PYV3 · SDP] ( 15 nm) are related to the torsion angle between the linker and the pyridine. Indeed, it directly relates to the π-conjugation between both rings of PYV3. For PYV3 · SDP, the torsion angles of the enol and keto forms are close to ${}^{\circ }$0 (${}^{\circ }$2 and ${}^{\circ }$3, respectively) which, compared to the PYV3 crystal values of ${}^{\circ }$30, means a greater π-conjugation and thus lower excitation energies. The opposite is true for PYV3-O [PYV3 · I3F3] where its torsion angles are larger than for PYV3: ${}^{\circ }$45 and ${}^{\circ }$35 for the enol and keto forms, respectively. For the other co-crystals, there is no direct relationship between the variations of the torsion angles compared to PYV3 [PYV3] and those, smaller, of the wavelengths of excitation.

### 2.2. Effects of the Environment

The effects of the environment are added in two steps: firstly, by considering the heteromers instead of the monomers in order to account for specific intermolecular interactions, then by further adding a point-charge embedding that reproduces the Ewald potential of the crystal, in order include the crystal field polarization. These calculations allow for the study of the direct effects of co-crystallization on the optical properties. For illustrative purposes, this section limits itself to two co-crystals, PYV3 · SA and PYV3 · FA1, which are isostructural. This will allow assessing whether the explicit and implicit crystal field effects display differences or not, despite their isostructurality.

#### 2.2.1. Inclusion of the Coformers

**Figure 4:**
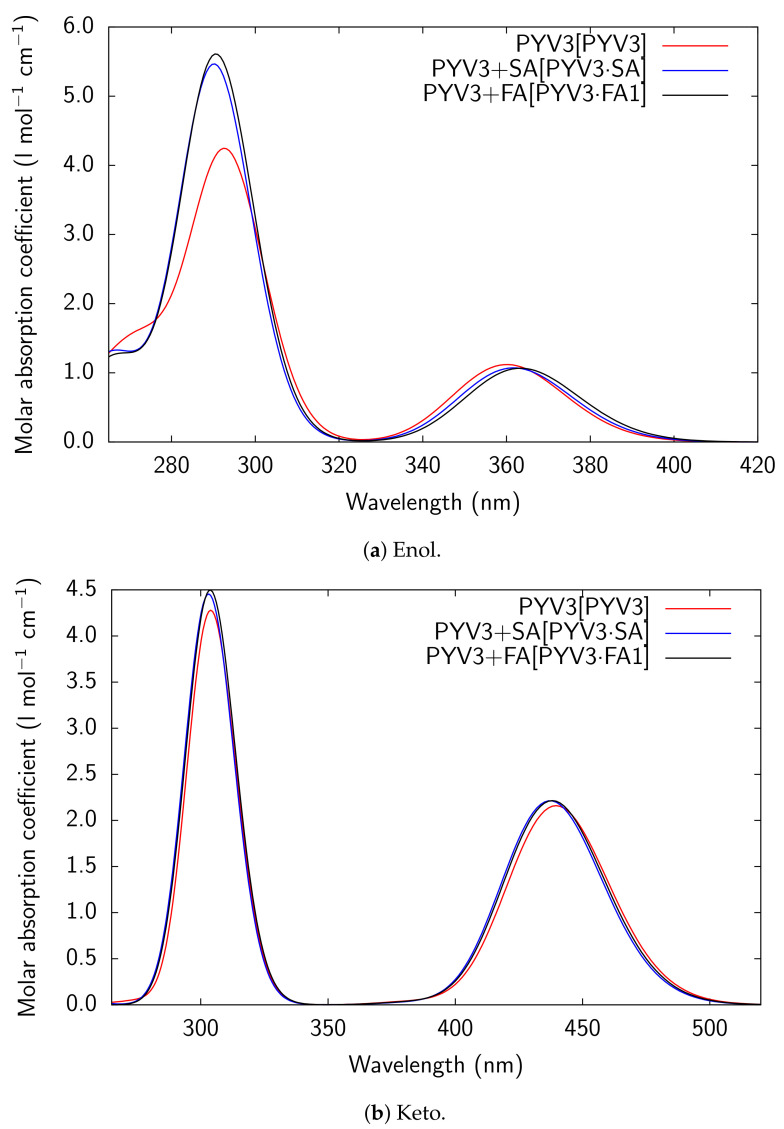
RI-CC2/def2-TZVPD UV/Vis absorption spectra of the isolated PYV3 [PYV3] and of the isolated heteromers of PYV3 · SA and PYV3 · FA1 co-crystals (PYV3 + SA [PYV3 · SA] and PYV3 + FA [PYV3 · FA1]).

By studying heteromers instead of monomers, i.e., PYV3 + SA [PYV3 · SA] and PYV3 + FA [PYV3 · FA1], both the geometry effects previously discussed and the electronic effects of the coformer are considered simultaneously. Table 2 gives the 1st excitation data for the heteromers of PYV3 · SA and PYV3 · FA1in comparison to PYV3. By adding the coformers, the first absorption band is slightly red-shifted for the enol forms (by ∼2 nm) and blue-shifted for the keto (by ∼1 nm) compared to PYV3 [PYV3]. The effect on the oscillator strength is small with variations of just a few percent with respect to PYV3: from -5–3. Looking at the simulated spectra, Figure 4, the enol heteromers of both PYV3 · SA and PYV3 · FA1 show more intense 2nd bands due to larger oscillator strengths for the second excited state (by 30, Table A6). The said bands are also slightly blue-shifted (by 2 nm) as the 2nd excitations of PYV3 · SA and PYV3 · FA1 are blue-shifted. In the case of PYV3 · FA1, the 3rd excitation is forbidden in favor of the 4th one that presents the same character as the corresponding excitation for PYV3 · SA (see later the discussion on the NTOs). For the keto forms, the effects of the geometry and of the inclusion of the coformer cancel each others for the 2nd bands (3rd excited states), so that they appear as unaffected (Figure 4, Table 2 and Table A7).

#### 2.2.2. Inclusion of the Crystal Field

**Figure 5:**
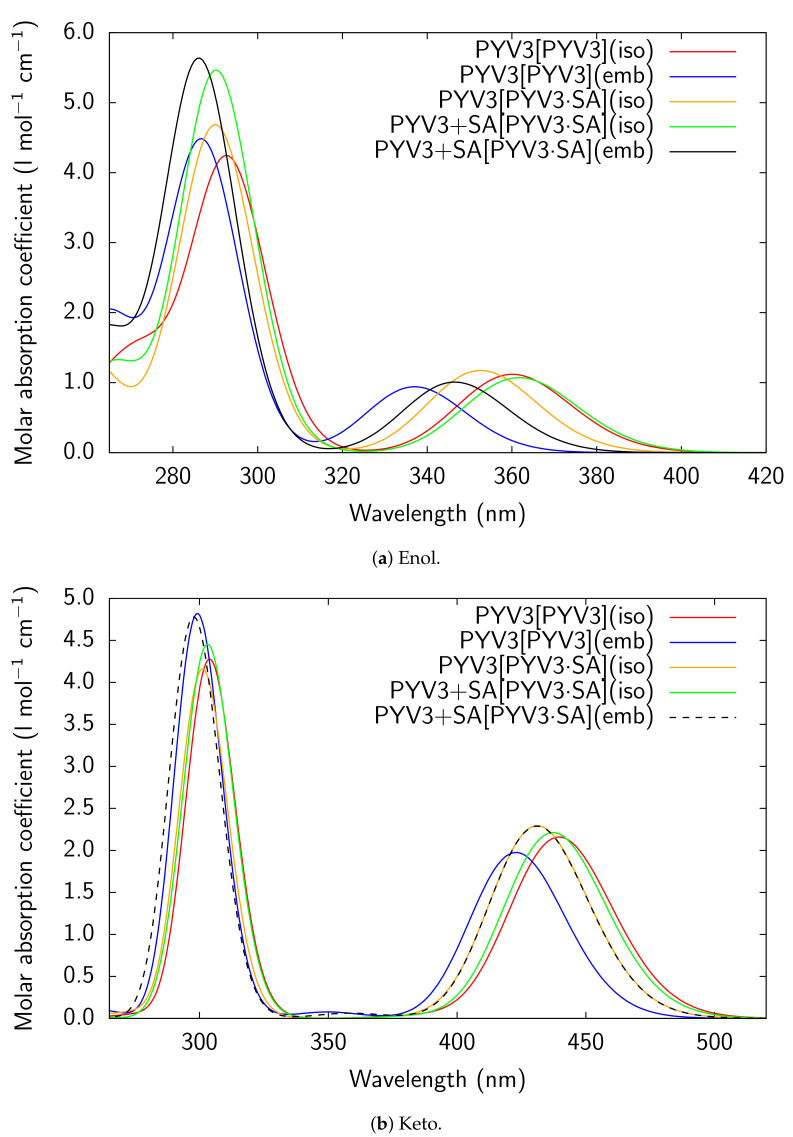
RI-CC2/def2-TZVPD UV/Vis absorption spectra of all models considered for PYV3 and PYV3 · SA.

The final step in this stepwise procedure for including the effects of the environment is to add an embedding of point-charges fitted to reproduce the crystalline Coulombic potential. Table 3 gives the 1st excitation data for the isolated and embedded forms of PYV3 and the variations for the embedded heteromers of the PYV3 · SA and ·FA1 co-crystals. Starting with the effect of the embedding on PYV3, the first excitation energy of both forms increases, blue-shifting the 1st absorption band by 23 nm for the enol and 17 nm for the keto. On the other hand, the oscillator strengths decrease, by  18 for the enol and 14 for the keto forms. The spectra, Figure 5, show that the second bands are also blue-shifted but more intensively. This is due to the increase of both the excitation energies and the intensities of the excited states contributing to the 2nd bands, see Table A8 and Table A9.

Figure 5 shows the UV/Vis absorption spectra of the various models of simulation for PYV3 and its co-crystal with SA. As just discussed for PYV3, the inclusion of the crystal field increases the excitation energies. This is also the case for PYV3 + SA [PYV3 · SA] when going from the isolated model to the embedded one for the two absorption bands of both enol and keto forms. For the enol, the 1st excitation is shifted by −16 nm ( 0.1515 eV), the 2nd by −4 nm ( 0.06 eV), and the 3rd by −2 nm ( 0.03 eV) with respect to the isolated molecule, i.e., PYV3 [PYV3] (Table 3 and Table A8). For the keto form, the variation on the first excitation is smaller than for the enol: −6 nm ( 0.04 eV). Its 2nd band, previously described by one excitation, is now defined by two almost degenerated excitations, the 3rd and 4th ones, at  −4 nm ( 0.05 eV) and −11 nm ( 0.15 eV) with respect to the 3rd excitation of the isolated heteromer, respectively (Table A9). The nature of these particular excitations is discussed in the next Section.

Compared to the embedded PYV3, the first absorption band of the co-crystal is red-shifted while the second one is unshifted. For the enol, the first band is of similar intensity while the second one is more intense. This is due to the larger oscillator strength of the 2nd excitation, despite a smaller value for the 3rd one. By opposition, the first band of the keto gets more intense than in PYV3 [PYV3] while the second one is similar (despite the dual excitation in the band). After embedding, the excitation energies of PYV3 · SA and PYV3 · FA1 remain very similar, with maximum differences of −2 nm, showing the extent of the effects of isostructurality (Figure A3).

### 2.3. Analysis of the NTOs

**Figure 6:**
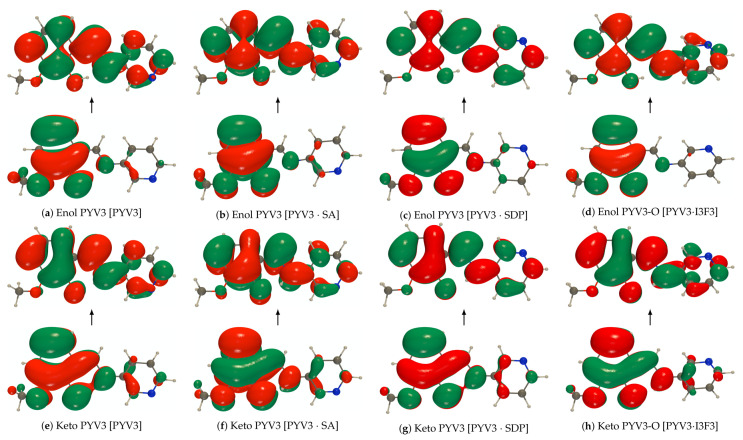
RI-CC2/def2-TZVPD NTOs (holes below the arrows and particles above them, the red (green) lobes are the positive (negative) ones) of the first excited state of isolated monomers of PYV3, PYV3 · SA, PYV3 · SDP, and PYV3·I3F3 (isovalue of 0.02 a.u.).

Concerning the NTOs, which characterize the nature of the electronic transitions, there are not affected by the changes in geometry. Figure 6 shows the NTOs of the enol and keto forms of the 1st excited states for the isolated PYV3 [PYV3], PYV3 [PYV3 · SA], PYV3 [PYV3 · SDP], and PYV3-O [PYV3 · I3F3]. All holes have the same π topology localized on the salicylidene part while all particles are the same, being of π character, fully delocalized over the molecule. As previously mentioned, the 3rd excited state for the enol form of the isolated PYV3 + FA [PYV3 · FA1] is actually forbidden in favor of the 4th one. Despite this change, the NTOs of that excitation are almost identical to those of the 3rd excited state of PYV3 [PYV3] (Figure A4). They differ by a smaller contribution of the pyridine to the hole. The same goes for the embedded PYV3 + FA [PYV3 · FA1] 4th excited state. When adding the effect of the crystal field to PYV3 [PYV3], the NTOs are unaffected for both the enol and keto forms, as seen in Figure A5 and Figure A6, respectively.

**Figure 7:**
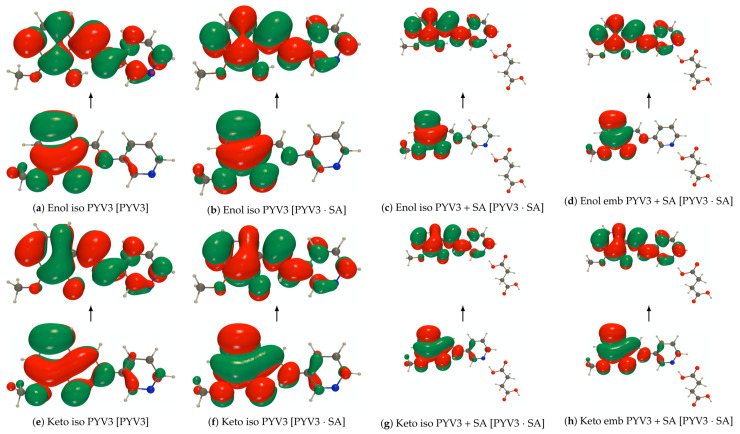
RI-CC2/def2-TZVPD NTOs (holes below the arrows and particles above them, the red (green) lobes are the positive (negative) ones) of the first excited state of isolated PYV3 [PYV3] and the various models of PYV3 · SA (isovalue of 0.02 a.u.). “Iso” stands for isolated while “emb" for embedded in crystal field point-charges.

**Figure 8:**
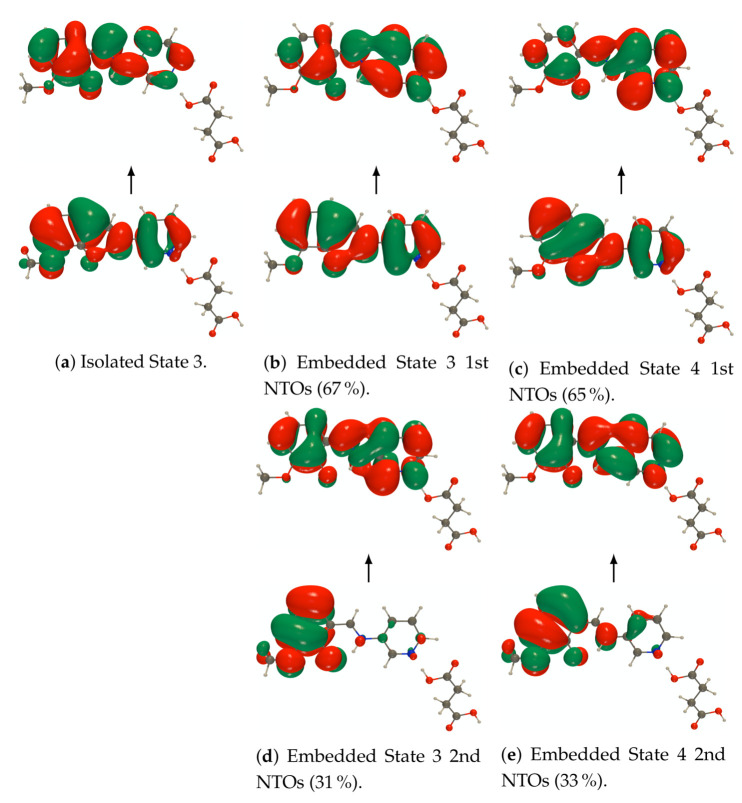
RI-CC2/def2-TZVPD NTOs (holes below the arrows and particles above them, the red (green) lobes are the positive (negative) ones) of the 3rd isolated keto form of PYV3 + SA [PYV3 · SA] and of the 3rd and 4th excited states of the embedded one (isovalue of 0.02 a.u.).

For all the models considered for the SA and FA1 co-crystals, the NTOs are almost not affected (Figure 7) except for the 3rd excitation of the embedded keto forms. Despite those few changes, all natural transition orbitals are localized on PYV3, i.e., the coformer polarizes PYV3 but does not directly contribute to the actual excitation. As previously mentioned, upon inclusion of crystal field, the 3rd excited state of the keto form of PYV3 + SA [PYV3 · SA] becomes degenerated into two excited states (the same goes for PYV3 + FA [PYV3 · FA1]). Each excitation is described by two pairs of NTOs, where the first one contributes to about 2/3 of the excitation and the second one to the remaining third (Figure 8). The holes of the first pair of NTOs have almost the same π topology as the isolated ones. For the second pairs, the holes are localized on the salicylidene part of the molecule and are of π nature. All the particles are similar to that of the isolated system. They differ by smaller contributions of the salicylidene part of the molecule for the first pair and increased σ character for the second pair.

### 2.4. Looking for a Computationally More Efficient Method

One limitation of this computational scheme is its cost for large heteromers, e.g., PYV3 + 2 × SDP [PYV3 · SDP] or PYV3-N + PYV3-O + 2 × I3F3 [PYV3 · I3F3], when employing the extended def2-TZVPD basis set.

**Figure 9:**
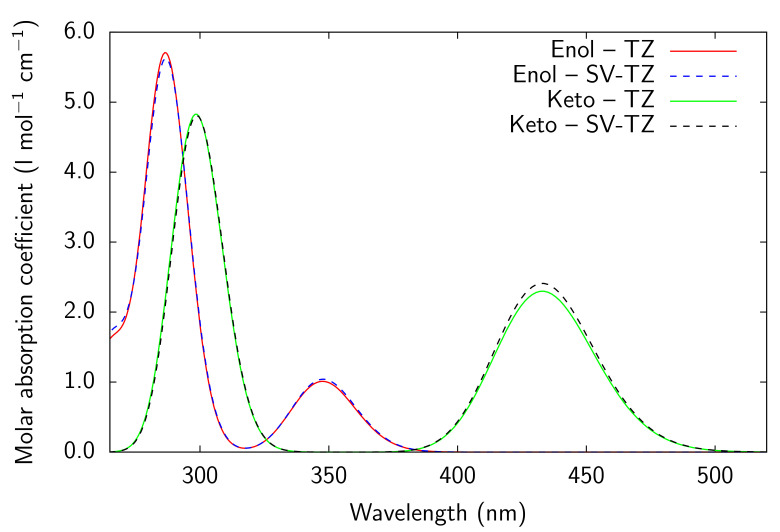
RI-CC2 composite (SV-TZ, dashed lines) and def2-TZVPD (TZ, full lines) spectra for the embedded heteromers of PYV3 · SA and PYV3 · FA1.

To circumvent this issue, we propose a composite technique where the effects of the coformer on the wavelengths of excitation are computed with the def2-SVPD basis set and added to the def2-TZVPD wavelengths of the monomer (Equation (1)). Alternatively, the same quantities can be seen as the def2-SVPD heteromer wavelength with a basis set correction on the molecule contributing most to the spectra (Equation ()).

(1) $$\begin{array}{cc} \lambda^{\mathrm{TZ}}\left(\mathrm{hetero}\right)\approx \lambda^{\mathrm{SV}-\mathrm{TZ}}\left(\mathrm{hetero}\right) & =\lambda^{\mathrm{TZ}}\left(\mathrm{mono}\right)+\left[\lambda^{\mathrm{SV}}\left(\mathrm{hetero}\right)-\lambda^{\mathrm{SV}}\left(\mathrm{mono}\right)\right] \end{array}$$

(2) $$\begin{array}{cc} \; & =\lambda^{\mathrm{SV}}\left(\mathrm{hetero}\right)+\left[\lambda^{\mathrm{TZ}}\left(\mathrm{mono}\right)-\lambda^{\mathrm{SV}}\left(\mathrm{mono}\right)\right] \end{array}$$

where TZ refers to a def2-TZVPD calculation and SV to a def2-SVPD one. In Table 4, the def2-TZVPD data for the excitations with non-negligible oscillator strengths are given for the embedded heteromers of PYV3 · SA and PYV3 · FA1 alongside the composite scheme values and its absolute errors. Overall, the composite scheme underestimates the excitation energies, but with errors that do not exceed 0.2 nm ( 0.005 eV) and as low as 0.02 nm ( 0.0003 eV). It is therefore safe to say that the composite scheme is accurate. Note that the errors on the keto excited state energies are smaller by about a factor two than the enol ones. The oscillator strengths obtained with def2-SVPD are comparable to the def2-TZVPD ones (Table 4), with errors that do not exceed 0.02. The composite spectra and the real def2-TZVPD ones are shown in Figure 9. As expected by the errors previously discussed, the spectra overlap almost perfectly. Furthermore, the def2-SVPD NTOs are identical to the def2-TZVPD ones (Figure A7 and Figure A8). A further step would be to use the embedded monomer RI-CCSD/def2-TZVPD excitation energies as a reference instead of the RI-CC2 ones.

## 3. Further Discussions, Conclusions, and Perspectives

This work aims at deciphering the effects of co-crystallization on the optical properties of PYV3, a *N*-salicylideneaniline derivative. To do so, a two step multi-scale method has been established. First the molecular geometry and the unit cell parameters are optimized using periodic boundary conditions density functional theory. Then, an embedding method is used to compute the RI-CC2 excitation energies and oscillator strengths. As a first step to compute the RI-CC2 optical properties, a basis set amongst the Ahlrichs family was selected: def2-TZVPD. It reproduces the results of the larger def2-QZVP basis set. Then, the accuracy of RI-CC2 is confirmed by comparing its excitation energies with RI-CCSD results. Indeed, despite small differences in the excitation energies with respect to RI-CCSD, RI-CC2 recovers the nature of the excitations. Then, in order to decipher the various effects of co-crystallization on the optical properties, the excitation properties and UV/Vis absorption spectra of isolated anil molecules as extracted from the co-crystals are computed. The changes of the geometry of the chromophore in the co-crystals lead to various effects on the excited states contributing to the UV/Vis absorption spectra. While for most coformers this results in negligible effect or in an increase of the excitation energies (occasionally accompanied by variations of the absorption intensities), PYV3 · SDP is the only one showing a significant decrease of the excitation energy. This has been attributed to the planarization of PYV3 in the PYV3 · SDP co-crystal. Then, the effects of the environment are included in two steps: (i) considering heteromers, i.e., the anil and its directly interacting coformer and (ii) including the crystal field thanks to an embedding of point-charges that reproduce the full Coulombic potential of the crystal. Combining both aspects of the environment shows that the PYV3 · SA co-crystal has lower first excitation energies compared to the pure crystal of PYV3. Analyzing these variations in detail, we have shown that the presence of the succinic acid coformer counteracts the hypsochromic shift due to the geometry and that crystal field effect leads to hypsochromic shifts of about 10 nm. Similar results have then been obtained for the isostructural PYV3 · FA1 co-crystal, showing that the geometry and the crystal field effects are negligibly modified by the difference in chemical nature of the coformer, succinic versus fumaric acid.

Based on these calculations of the vertical excitation energies of PYV3 and its co-crystals with SA and FA, comparisons have been drawn with experiment, where the onsets of absorption for the enol and keto forms have been determined from the spectra of Ref. [1]. For the enol form, this onset of absorption goes from 460 nm in the case of PYV3 to 465 nm and 475 nm for PYV3 · SA and PYV3 · FA1 in comparison to 337 nm, 346 nm, and  348 nm, respectively, demonstrating the good qualitative agreement. In addition, for the keto form, the calculated values amount to 423 nm, 431 nm, and  433 nm in comparison to experimental values of 560 nm, 565 nm, and  575 nm, for PYV3, PYV3 · SA, and PYV3 · FA1, respectively. Though the small co-crystallization-induced hypsochromic shifts are predicted for PYV3 · SA and PYV3 · FA1 by the two-step multi-scale method, the absorption spectra remain similar and the nature of the transitions, as revealed by an NTO analysis, is not affected by co-crystallization. Thus, at this level of investigation, there is no evidence to explain the difference in the photochromic behavior of the PYV3 crystal compared to the PYV3 · SA and PYV3 · FA1 co-crystals. Studying the excited state dynamics appears necessary to address this effect, which goes beyond the aim of the present work.

Finally, a composite scheme to obtain accurate but computationally more accessible UV/Vis absorption spectra has been established, and proven to be sucessful in recovering the key features of the excitations. In view of future investigations, we note that other approaches can be explored such as other charge definitions (electrostatic potential, ESP, charges or natural bond orbital, NBO, charges), self-consistent embeddings, or extracting the excitation energies from frequency dispersion fits of the polarizability (available for selected DFT functionals in CRYSTALmathsizesmall17 [23]).

## 4. Methods and Computational Aspects

Crystal geometries were taken from Ref. [2]. They have been obtained from full geometry optimizations (geometry of the molecules and unit cell parameters) performed using periodic boundary conditions (PBC) density functional theory (DFT) calculations, as implemented in the CRYSTALmathsizesmall14 package [24]. Previously, we have shown that the PBEsol0 exchange-correlation functional (the global hybrid variant of PBEsol [25]) used with Pople’s 6-31G(d,p) (as taken from the Basis Set Exchange website [26,27,28]) is appropiate for the optimization of crystalline anils [29,30]. For iodine, the LANL2DZ effective core potential (ECP) was used. Default convergence parameters were used while the shrinking factor of the irreducible Brillouin zone was set to 6 (yielding between 64 and 112 points of integration) and the tolerance criteria for the Coulomb and exchange integrals (keyword TOLINTEG) was set to “8 8 8 8 16”.

The Mulliken charges were evaluated with the same method as for the crystal geometry optimizations. Using those charges, the Ewald program [7,8] (modified to handle partial charges) was used to generate arrays of point-charges that reproduce the full Coulombic potential of the crystal. The parameters used for each crystal are given in the Supporting Information, Table A1.

Vertical excitation energies and oscillator strengths were computed using the coupled cluster approximate doubles (CC2) [9] and coupled cluster singles and doubles (CCSD) models in combination with the resolution of the identity (RI) approximation [10,11] (RI-CC2 and RI-CCSD) with Ahlrichs and coworkers’ basis sets [31,32,33,34] as implemented in the TURBOMOLEmathsizesmall package [35]. The DrawSpectrum program from the DrawMol suite [36] was then used to transform the excited state data into UV/Vis absorption spectra using Gaussian functions with a full width at half maximum value of 0.3 eV.

To analyze the excitations, the natural transition orbitals (NTOs) were computed by TURBOMOLEmathsizesmall [35] and then represented using DrawMol [36]. An excited state can be described by a huge combination of simple determinant-based electronic transitions, which are given in terms of the MOs. The transition density matrix of single excitations, T, thus takes the dimensions $\left(N_{o},N_{u}\right)$, where $N_{o}$ is the number of occupied MOs and $N_{u}$ the number of unoccupied MOs. By applying unitary transformations to T, it can be reduced to a square matrix of dimension $\left(N_{o},N_{o}\right)$. This effectively condenses the information of the transitions into a smaller set of orbitals, the NTOs, that describe the hole created by the electronic promotion (in the occupied space) and the corresponding created particle (in the unoccupied space) [17,18].

### 4.1. Effects of the Basis Set

**Figure 10:**
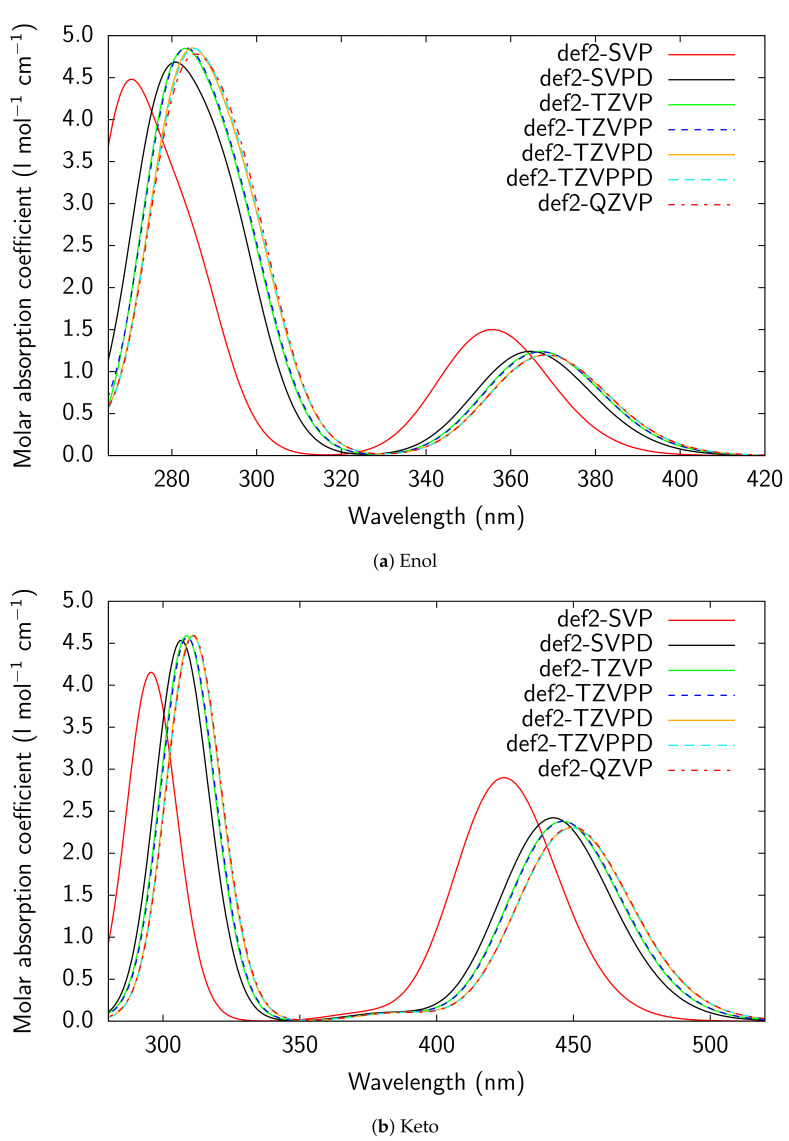
RI-CC2 UV/Vis absorption spectra of the isolated enol and keto heteromers of PYV3 · I2but, PYV3 + I2but [PYV3 · I2but], as a function of the basis set.

In order to select a basis set, the UV/Vis absorption spectrum of both forms of the PYV3 · I2but heteromer (PYV3 + I2but [PYV3 · I2but]) were simulated for a broad selection of Ahlrichs basis sets (from a split valence set, def2-SVP, to a quadruple-ζ one, def2-QZVP), see Figure 10. The composition of the considered basis sets are given in Table A2. For iodine, the def-ECP effective core potential was used. Both enol and keto spectra present two absorption bands, at about 365 nm and 290 nm for the enol and 450 nm and 310 nm for the keto forms. As the basis set becomes larger (and thus more complete), the excitation energies decrease and the bands are red-shifted. Since the def2-TZVPD, def2-TZVPPD, and def2-QZVP spectra overlap perfectly, the smallest of three equally performing sets, def2-TZVPD, is, therefore, a suitable basis set for further calculations on the co-crystals. In order to confirm the selection of def2-TZVPD, the 1st excitation energies, oscillator strengths, and their variations with respect to the largest considered set for both forms of the I2but heteromer are given in Table 5. With errors of the order of 0.1 nm for the wavelengths (corresponding to 0.001 eV on the excitation energies) and 0.001 for the oscillator strengths, def-TZVPD yields converged results with respect to def2-QZVP. Unless stated otherwise, all the following calculations were performed with the def2-TZVPD basis set.

### 4.2. RI-CC2 vs. RI-CCSD

In order to evaluate the error made by using RI-CC2, isolated phase RI-CCSD excitation energy calculations on the monomers of PYV3 (PYV3 [PYV3]) have been performed, see Table 6. Note that RI-CCSD oscillator strengths are not available in TURBOMOLEmathsizesmall[35] and thus can not be compared to the RI-CC2 ones. For the first five excited states, RI-CC2 systematically underestimates the excitation energy. The error made is state-dependent, e.g., the error for excited state 1 of the enol form amounts to 37 nm ( 0.30 eV), while for state 2, the error is 17 nm ( 0.25 eV). This leads to the following average errors and standard deviations: 19(10) nm for the enol and 29(20) nm for the keto forms. In other words, to recover the RI-CCSD spectra, the RI-CC2 cannot simply be shifted. The difference between the two methods is due to the different contributions of the singles and doubles clusters, $T_{1}$ and $T_{2}$, to the excited states between the RI-CC2 and RI-CCSD results, see Table A3. Indeed, the contribution of $T_{1}$ to an excited state relates to the character of the excitation. For RI-CC2 calculations, $T_{1}$ strongly contributes to all 5 calculated excited states, ∼88, meaning that they have a single excitation character. When going to RI-CCSD, the $T_{1}$ contributions further increase to ∼92, thus confirming the singles character. [37,38] Had one of the excitations actually been of double character, the $T_{1}$ contribution would have decreased when going from RI-CC2 (which does not explicitly allow for double excitations [9]) to RI-CCSD.

**Figure A1:**
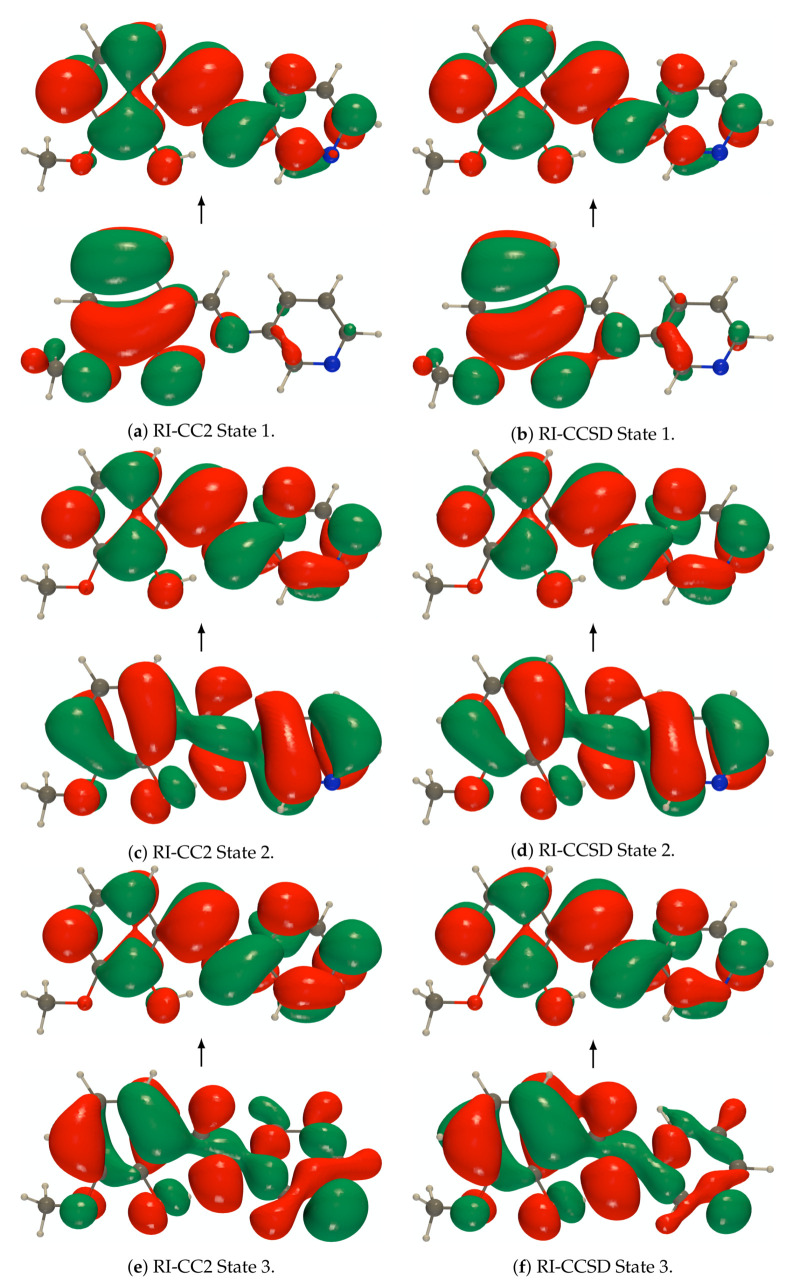
def2-TZVPD NTOs (holes below the arrows and particles above them, the red (green) lobes are the positive (negative) ones) of the isolated PYV3 [PYV3] enol form (isovalue of 0.02 a.u.).

**Figure A2:**
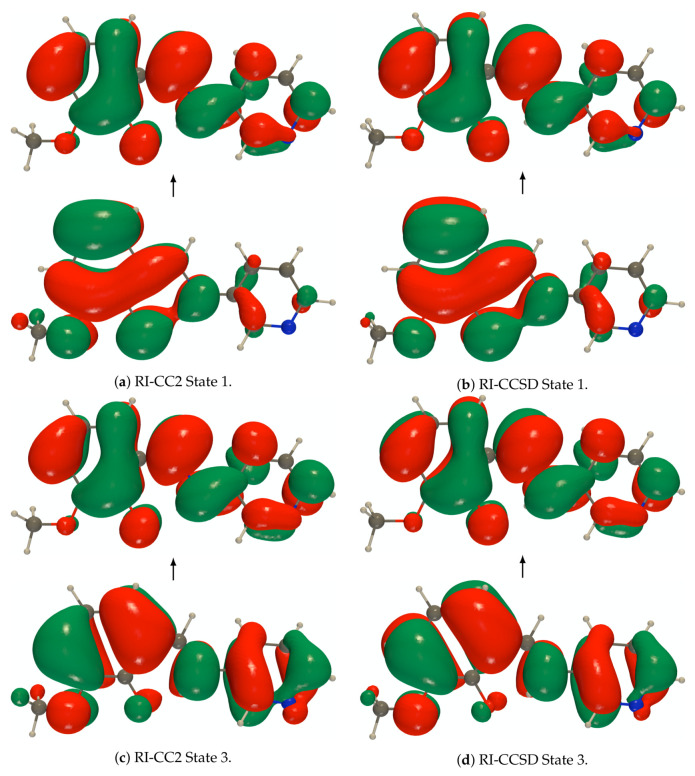
def2-TZVPD NTOs (holes below the arrows and particles above them, the red (green) lobes are the positive (negative) ones) of the isolated PYV3 [PYV3] keto form (isovalue of 0.02 a.u.).

**Figure A3:**
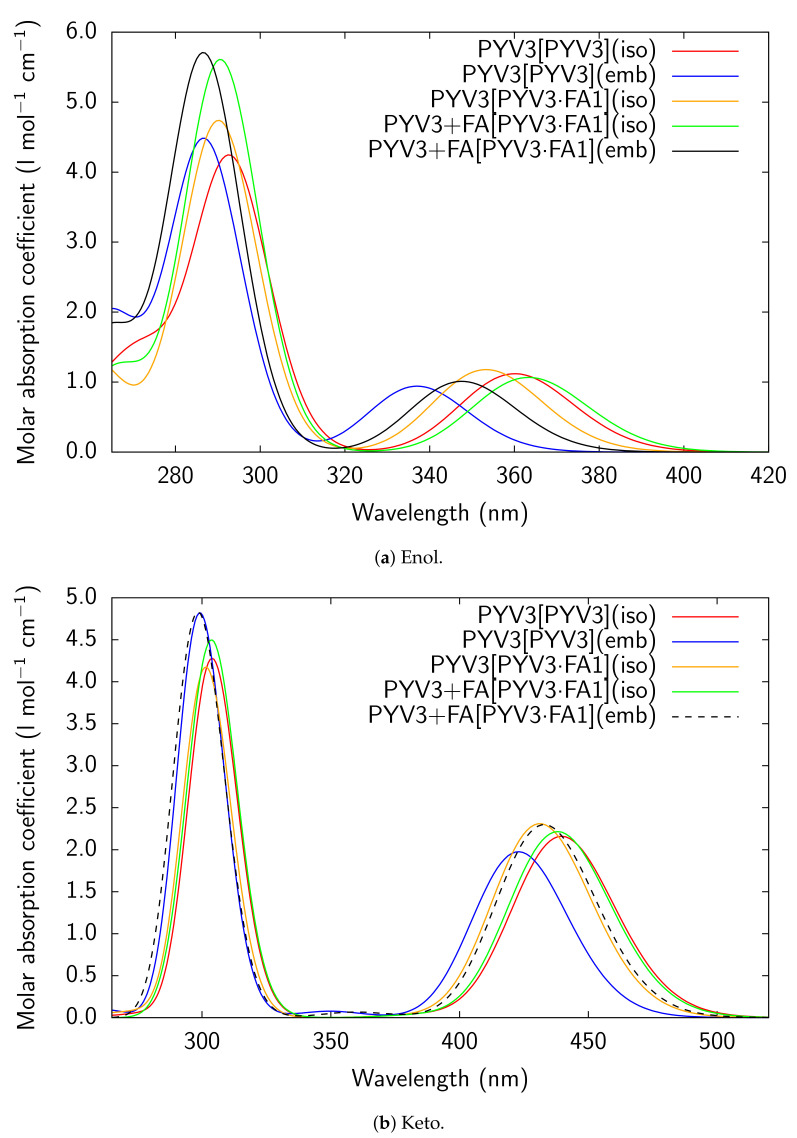
RI-CC2/def2-TZVPD UV/Vis absorption spectra of all models considered for PYV3 and PYV3 · FA1.

**Figure A4:**
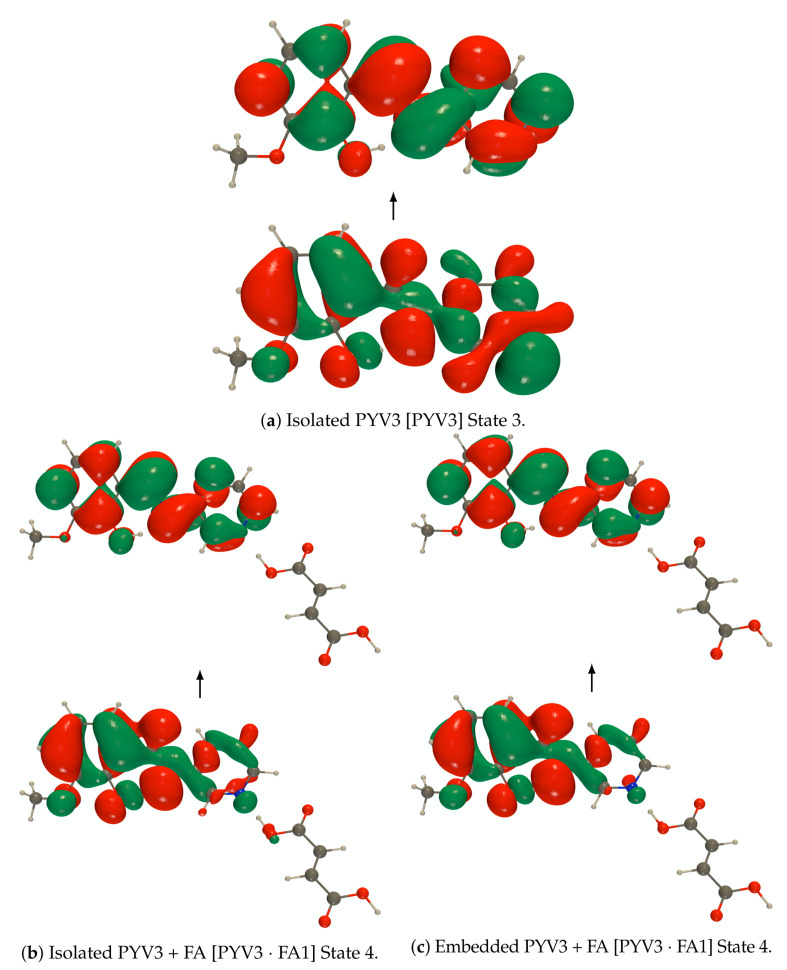
RI-CC2/def2-TZVPD NTOs (holes below the arrows and particles above them, the red (green) lobes are the positive (negative) ones) of the 3rd excited state of the isolated PYV3 [PYV3] enol form and of the 4th excited state of the isolated and embedded PYV3 + FA [PYV3 · FA1] (isovalue of 0.02 a.u.).

**Figure A5:**
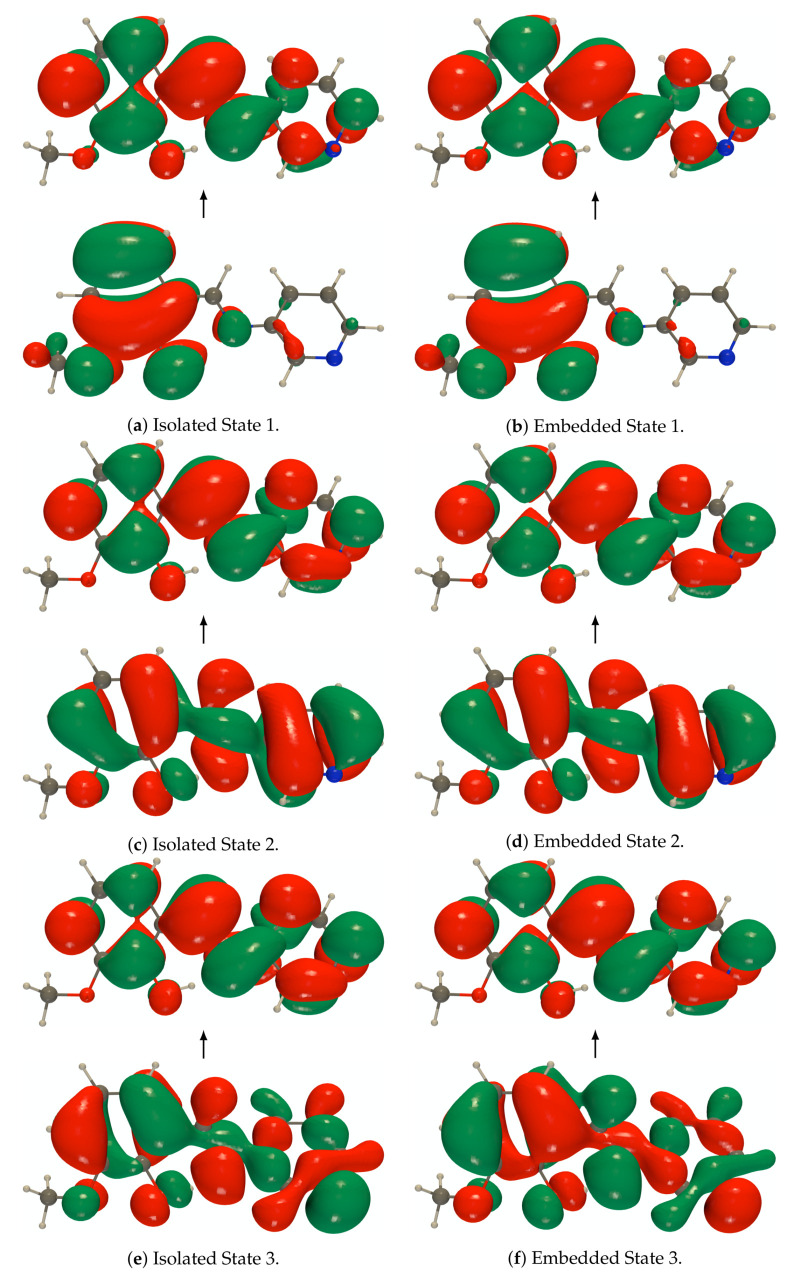
RI-CC2/def2-TZVPD NTOs (holes below the arrows and particles above them, the red (green) lobes are the positive (negative) ones) of the isolated and embedded PYV3 [PYV3] enol form (isovalue of 0.02 a.u.).

**Figure A6:**
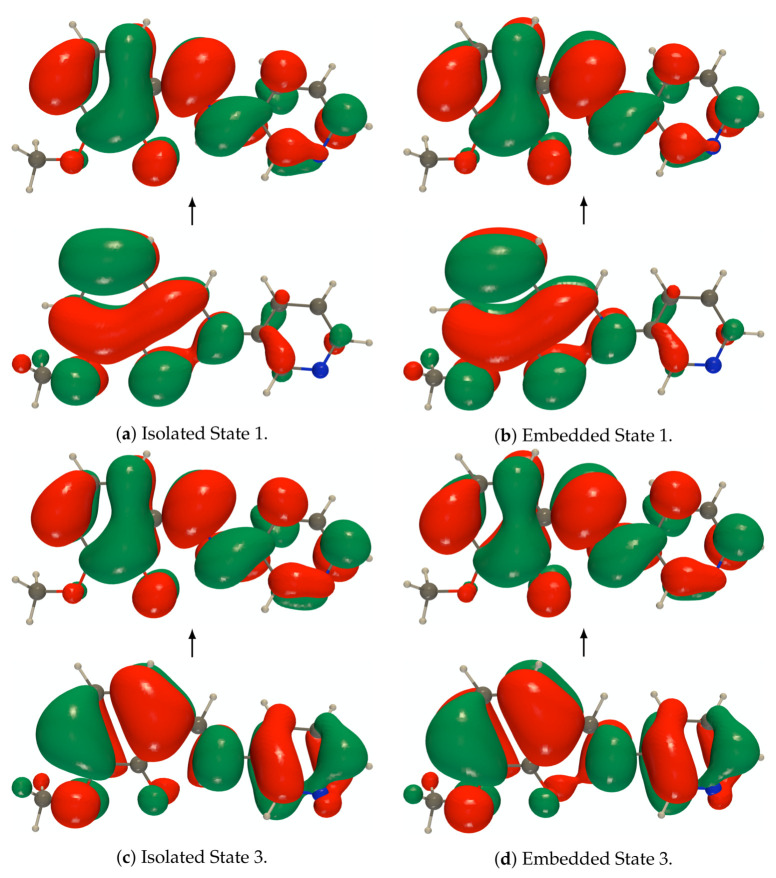
RI-CC2/def2-TZVPD NTOs (holes below the arrows and particles above them, the red (green) lobes are the positive (negative) ones) of the isolated and embedded PYV3 [PYV3] keto form (isovalue of 0.02 a.u.).

**Figure A7:**
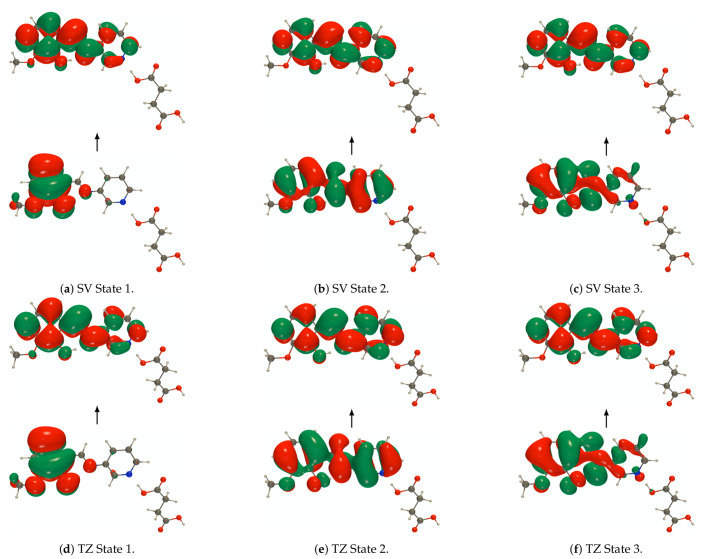
RI-CC2 def2-SVPD (SV) and def2-TZVPD (TZ) NTOs (holes below the arrows and particles above them, the red (green) lobes are the positive (negative) ones) of the embedded PYV3 + SA [PYV3 · SA] enol form (isovalue of 0.02 a.u.).

**Figure A8:**
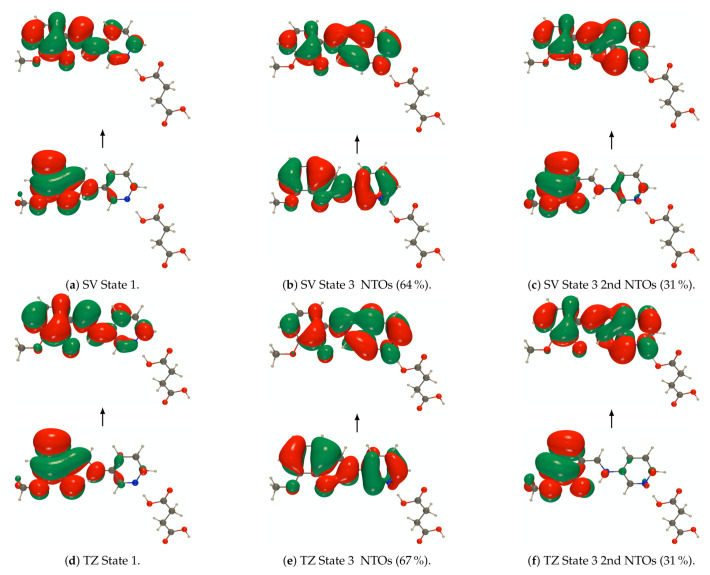

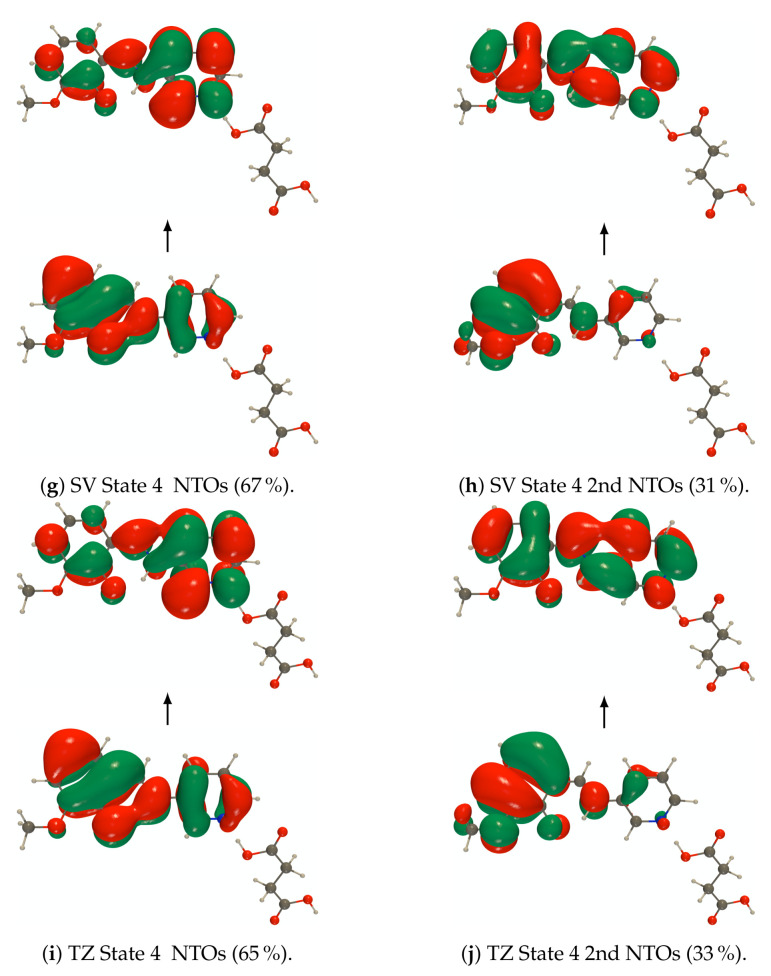
RI-CC2 def2-SVPD (SV) and def2-TZVPD (TZ) NTOs (holes below the arrows and particles above them, the red (green) lobes are the positive (negative) ones) of the embedded PYV3 + SA [PYV3 · SA] keto form (isovalue of 0.02 a.u.).

The RI-CC2 NTOs of the enol form perfectly match the RI-CCSD ones, see Figure A1. All particles are purely of π nature while from states 1 to 3, purely π holes become increasingly mixed with some σ character: for state 2, a σ part is on the double bond and for state 3 on the pyridine part (right-hand side). The largest difference between the two methods is for the 3rd excited state: the hole NTO for RI-CCSD is less localized on the pyridine part than for RI-CC2, see Figure A1e,f. On the other hand, the corresponding particles are identical. For the keto form, the NTOs of the RI-CC2 and RI-CCSD excited states are the same, where both holes and particles are of π nature, see Figure A2. Overall, RI-CC2 recovers the features of the excitations calculated with RI-CCSD, the main difference being energy shifts that are excited state-dependent.

## Figures and Tables

**Table 1:** RI-CC2/def2-TZVPD first wavelength of excitation (λ, in nm) and oscillator strength (*f*) for the isolated monomers of PYV3 (PYV3 [PYV3]) and their variations upon co-crystallization (PYV3 [PYV3 · XXX]).

	Enol		Keto	
Crystal	λ	*f*	λ	*f*
**PYV3 [PYV3]**	**360**	**0.125**	**440**	**0.240**
PYV3 [PYV3 · SA]	−7	+5%	−8	+6%
PYV3 [PYV3 · FA1]	−7	+5%	−8	+7%
PYV3 [PYV3 · FA2]	0	+3%	−7	+0%
PYV3 [PYV3 · DHBP]	−1	+6%	−3	+0%
PYV3 [PYV3 · SDP]	+15	+14%	+14	+8%
PYV3 [PYV3 · I2but]	−2	+4%	+2	+2%
PYV3 [PYV3 · I2F4]	+2	+6%	0	+2%
PYV3-N [PYV3 · I3F3]	−18	−13%	−12	+17%
PYV3-O [PYV3 · I3F3]	−27	−25%	−29	+11%

**Table 2:** RI-CC2/def2-TZVPD first excitation energy (λ, in nm) and oscillator strength (*f*) for the isolated monomer of PYV3 (PYV3 [PYV3]) and the variations for the heteromers of co-crystals (PYV3 + XXX [PYV3 · XXX]).

	Enol		Keto	
	λ	f	λ	f
**PYV3 [PYV3]**	**360**	**0.125**	**440**	**0.240**
PYV3 + SA [PYV3 · SA]	+2	−4%	−2	+2%
PYV3 + FA [PYV3 · FA1]	+3	−5%	−1	+3%

**Table 3:** RI-CC2/def2-TZVPD first wavelengths of excitation (λ, in nm) and oscillator strengths (*f*) for the isolated (iso) and embedded (emb) monomer of PYV3 (PYV3 [PYV3]) and the variations for the embedded heteromers of co-crystals (PYV3 + XXX [PYV3 · XXX], with respect to the embedded PYV3).

		Enol		Keto	
		λ	f	λ	f
PYV3 [PYV3]	(iso)	360	0.125	440	0.240
PYV3 [PYV3]	(emb)	337	0.105	423	0.220
PYV3 + SA [PYV3 · SA]	(emb)	+9	+7%	+8	+16 %
PYV3 + FA [PYV3 · FA1]	(emb)	+11	+7%	+10	+16%

**Table 4:** RI-CC2 wavelengths of excitation with the def2-TZVPD (TZ) and composite (SV-TZ) methods (λ, in nm) and def2-TZVPD (TZ) and def2-SVPD (SV) oscillator strengths, *f*, and absolute errors (AE) for the embedded heteromers of SA and FA1 (PYV3 + SA [PYV3 · SA] and PYV3 + FA [PYV3 · FA1]).

		Enol					
Co-Crystal	State	$\lambda^{\mathrm{TZ}}$	$\lambda^{\mathrm{SV}-\mathrm{TZ}}$	AE	$f^{\mathrm{TZ}}$	$f^{\mathrm{SV}}$	AE
PYV3 + SA	1	346.5	346.7	0.2	0.1122	0.1155	0.004
[PYV3 · SA]	2	286.2	286.4	0.2	0.6243	0.6155	-0.008
(emb)	3	264.2	264.4	0.2	0.1670	0.1780	0.011
PYV3 + FA	1	347.6	347.8	0.2	0.1125	0.1158	0.003
[PYV3 · FA1]	2	286.7	286.8	0.1	0.6315	0.6221	-0.010
(emb)	4	265.0	265.2	0.2	0.1666	0.1781	0.011
		Keto					
PYV3 + SA	1	431.1	431.2	0.1	0.2547	0.2671	0.012
[PYV3 · SA]	3	300.2	300.3	0.1	0.3602	0.3726	0.013
(emb)	4	293.2	293.3	0.1	0.2099	0.1927	-0.017
PYV3 + FA	1	432.9	433.0	0.1	0.2557	0.2682	0.012
[PYV3 · FA1]	3	301.3	301.4	0.1	0.3329	0.3453	0.012
(emb)	4	294.4	294.5	0.1	0.2444	0.2269	-0.017

**Table 5:** RI-CC2 first wavelength of excitation (λ, in nm) and oscillator strength (*f*), and their variations (Δλ and Δf) with respect to def2-QZVP for the isolated PYV3 · I2but [PYV3 · I2but] heteromer as a function of the basis set. All calculations were performed at the RI-CC2 level of approximation.

	Enol			
Basis Set	λ	Δλ	*f*	Δf
def2-SVP	355.7	−12.8	0.167	0.034
def2-SVPD	364.7	−3.7	0.138	0.005
def2-TZVP	367.0	−1.5	0.138	0.005
def2-TZVPP	367.2	−1.3	0.138	0.004
def2-TZVPD	368.3	−0.2	0.133	0.000
def2-TZVPPD	368.5	0.1	0.133	0.000
def2-QZVP	368.4	0.0	0.133	0.000
	Keto			
def2-SVP	424.7	−24.9	0.323	0.065
def2-SVPD	442.6	−6.9	0.269	0.012
def2-TZVP	446.0	−3.6	0.265	0.007
def2-TZVPP	446.3	−3.2	0.265	0.007
def2-TZVPD	449.4	−0.1	0.256	−0.001
def2-TZVPPD	449.6	0.1	0.256	−0.001
def2-QZVP	449.5	0.0	0.257	0.000

**Table 6:** Wavelengths of excitation of the first five excited states (in nm) and error (Δλ) and the average error and standard deviations (Std. Dev.) with respect to CCSD for the isolated forms of PYV3 [PYV3]. All calculations employ the RI approximation and the def2-TZVPD basis set.

	Enol				Keto		
State	CC2	CCSD	Δλ		CC2	CCSD	Δλ
1	360.1176718	323.5613853	36.56		439.5947873	403.6075165	35.99
2	292.9274818	276.3636451	16.56		386.0223205	324.858493	61.16
3	272.5035122	252.1121005	20.39		303.9156012	282.7609954	21.15
4	260.3818293	247.5070329	12.87		275.8809133	258.4915781	17.39
5	252.8999347	242.0757774	10.82		257.5112374	246.5659191	10.95
Average			19.44				29.33
Std. Dev.			10.24				20.03

## Appendix A

**Table A1:** Parameters used in the Ewald program.

Crystal	Zone 1	Number of Charges	Unit Cell Composition	Supercell	Total
	Composition	in Zone 2		“Zones 1 + 2 + 3”	Number
	(Number of Atoms )	(Diameter of Zone 2)		Dimensions	of Atoms ^a^
PYV3	PYV3 (29)	4971 (44 *Å*)	4 PYV3	16 × 10 × 4	74,240
PYV3 SA	PYV3 + SA (43)	9957 (56 *Å*)	4 PYV3 + 2 SA	20 × 4 × 10	115,200
PYV3 FA1	PYV3 + FA (41)	9959 (56 *Å*)	4 PYV3 + 2 FA	20 × 4 × 10	112,000

^a^ The total number of atoms corresponds to the sum of the numbers of atoms in zones 1, 2, and 3, in other words, the sum of the number of point charges in zones 2 and 3 and the number of atoms in the QC zone 1.

**Table A2:** Ahlrichs basis sets and their contracted compositions for the atoms in PYV3 · I2but.

Basis Set	Composition	
def2-SVP	C, N, O, F	3s 2p 1d
	I	4s 4p 2d
	H	2s 1p
def2-SVPD	C, N	4s 2p 2d
	O, F	4s 3p 2d
	I	5s 5p 3d
	H	2s 2p
def2-TZVP	C, N, O, F	5s 3p 2d 1f
	I	6s 5p 3d 2f
	H	3s 1p
def2-TZVPP	C, N, O, F, I	def2-TZVP
	H	3s 2p 1d
def2-TZVPD	C, N	6s 3p 3d 1f
	O, F	6s 4p 3d 1f
	I	7s 6p 4d 2f
	H	3s 2p
def2-TZVPPD	C, N, O, F, I	def2-TZVPD
	H	3s 3p 1d
def2-QZVP	C, N, O, F	7s 4p 3d 2f 1g
	I	7s 6p 4d 4f 1g
	H	4s 3p 2d 1f

**Table A3:** Contributions of the $T_{1}$ and $T_{2}$ clusters to the RI-CC2 and RI-CCSD excited states of the enol and keto forms of the isolated PYV3 [PYV3] molecule (in %), as calculated with the def2-TZVPD basis set.

	Enol			
	RI-CC2		RI-CCSD	
State	$T_{1}$	$T_{2}$	$T_{1}$	$T_{2}$
1	87.14	12.86	92.17	7.83
2	88.96	11.04	92.98	7.02
3	87.64	12.36	92.93	7.07
4	87.62	12.38	91.56	8.44
5	88.84	11.16	92.94	7.06
	Keto			
1	87.38	12.62	91.79	8.21
2	85.49	14.51	92.31	7.69
3	87.62	12.38	92.42	7.58
4	86.55	13.45	91.78	8.22
5	89.25	10.75	94.45	5.55

**Table A4:** RI-CC2/def2-TZVPD wavelengths of excitation, λ in nm, and oscillator strengths, *f*, for the enol PYV3 molecule in its crystal (PYV3 [PYV3]) and its co-crystals (PYV3 [PYV3 · XXX]). For PYV3 [PYV3], the absolute values are given while for the co-crystals, the absolute variations and relative variations are given for the wavelengths and oscillator strengths, respectively.

	2nd Absorption Band				
	2nd Excitation			3rd Excitation	
Crystal	λ	*f*		λ	*f*
PYV3 [PYV3]	293	0.466		273	0.144
PYV3 [PYV3 · SA]	−3	+10%		+3	−75%
PYV3 [PYV3 · FA1]	−2	+11%		+4	−73%
PYV3 [PYV3 · FA2]	+2	−24%		+2	+13%
PYV3 [PYV3 · DHBP]	−1	+8%		+4	−45%
PYV3 [PYV3 · SDP]	−2	+54%		—	
PYV3 [PYV3 · I2but]	+8	−75%		+9	+301%
PYV3 [PYV3 · I2F4]	+11	−72%		+10	+287%
PYV3-N [PYV3 · I3F3]	−4	−89%		0	+356%
PYV3-O [PYV3 · I3F3]	−11	−75%		−8	+267%

**Table A5:** RI-CC2/def2-TZVPD wavelengths of excitation, λ in nm, and oscillator strengths, *f*, for the keto PYV3 molecule in its crystal (PYV3 [PYV3]) and its co-crystals (PYV3 [PYV3 · XXX]). For PYV3 [PYV3], the absolute values are given while for the co-crystals, the absolute variations and relative variations are given for the wavelengths and oscillator strengths, respectively.

	2nd Absorption Band	
	3rd Excitation	
	λ	f
PYV3 [PYV3]	304	0.476
PYV3 [PYV3 · SA]	−3	−3%
PYV3 [PYV3 · FA1]	−2	−2%
PYV3 [PYV3 · FA2]	0	0%
PYV3 [PYV3 · DHBP]	0	0%
PYV3 [PYV3 · SDP]	+6	+4%
PYV3 [PYV3 · I2but]	+4	+1%
PYV3 [PYV3 · I2F4]	+3	+1%
PYV3-N [PYV3 · I3F3]	0	+1%
PYV3-O [PYV3 · I3F3]	−11	−3%

**Table A6:** RI-CC2/def2-TZVPD wavelengths of excitation, λ in nm, and oscillator strengths, *f*, for the enol forms of isolated PYV3 (PYV3 [PYV3]) and the heteromers of its co-crystals (PYV3 + XXX [PYV3 · XXX]). For PYV3 [PYV3], the absolute values are given while for the co-crystals, the absolute variations and relative variations are given for the wavelengths and oscillator strengths, respectively.

	2nd Absorption Band				
	2nd Excitation			3rd Excitation	
Crystal	λ	*f*		λ	*f*
PYV3 [PYV3]	293	0.466		273	0.144
PYV3 + SA [PYV3 · SA]	−3	+30%		−6	−8%
PYV3 + FA [PYV3 · FA1] ^a^	−2	+33%		−6	−12%

^a^ The oscillator strength of actual 3rd excited state is null, here the data is given for the 4th one.

**Table A7:** RI-CC2/def2-TZVPD wavelengths of excitation, λ in nm, and oscillator strengths, *f*, for the keto forms of isolated PYV3 (PYV3 [PYV3]) and the heteromers of its co-crystals (PYV3 + XXX [PYV3 · XXX]). For PYV3 [PYV3], the absolute values are given while for the co-crystals, the absolute variations and relative variations are given for the wavelengths and oscillator strengths, respectively.

	2nd Absorption Band	
	3rd Excitation	
Crystal	λ	*f*
PYV3 [PYV3]	304	0.476
PYV3 + SA [PYV3 · SA]	0	0%
PYV3 + FA [PYV3 · FA1]	0	0%

**Table A8:** RI-CC2/def2-TZVPD wavelengths of excitation (λ in nm) and oscillator strengths (*f*) for the enol forms of the isolated and embedded PYV3 (PYV3 [PYV3]) and the embedded heteromers of its co-crystals (PYV3 + XXX [PYV3 · XXX]). For PYV3 [PYV3], the absolute values are given while for the co-crystals the absolute variations and relative variations with respect to the embedded PYV3 are given for the wavelengths and oscillator strengths, respectively.

		2nd Absorption Band				
		2nd Excitation			3rd Excitation	
Crystal		λ	*f*		λ	*f*
PYV3 [PYV3]	(iso)	293	0.466		273	0.144
PYV3 [PYV3]	(emb)	287	0.495		264	0.214
PYV3 + SA [PYV3 · SA]	(emb)	−1	+26%		0	−22%
PYV3 + FA [PYV3 · FA1] ^a^	(emb)	0	+27%		0	−22%

^a^ The oscillator strength of actual 3rd excited state is null, here the data is given for the 4th one.

**Table A9:** RI-CC2/def2-TZVPD wavelengths of excitation (λ, in nm) and oscillator strengths (*f*) for the keto forms of the isolated and embedded PYV3 (PYV3 [PYV3]) and the embedded heteromers of its co-crystals (PYV3 + XXX [PYV3 · XXX]). For PYV3 [PYV3], the absolute values are given while for the co-crystals the absolute variations and relative variations with respect to the embedded PYV3 are given for the wavelengths and oscillator strengths, respectively.

		2nd Absorption Band				
		3rd Excitation			4th Excitation	
Crystal		λ	*f*		λ	*f*
PYV3 [PYV3]	(iso)	304	0.476		—	
PYV3 [PYV3]	(emb)	299	0.536		—	
PYV3 + SA [PYV3 · SA]	(emb)	+1	−33%		−6	−61%
PYV3 + FA [PYV3 · FA1]	(emb)	+2	−38%		−5	−54%
